# Efficacy of a Rinse Containing Sea Salt and Lysozyme on Biofilm and Gingival Health in a Group of Young Adults: A Pilot Study

**DOI:** 10.1155/2017/4056708

**Published:** 2017-12-19

**Authors:** Jeyaraj Hoover, Eduardo Tovar, Trevor Zlatnik, Chandima Karunanayake

**Affiliations:** ^1^College of Dentistry, University of Saskatchewan, 105 Wiggins Road, Saskatoon, SK, Canada; ^2^Canadian Centre for Health and Safety in Agriculture, University of Saskatchewan, 104 Clinic Place, Saskatoon, SK, Canada S7N 2Z4

## Abstract

**Objectives:**

To evaluate new mouth rinse containing sea salt, xylitol, and lysozyme on biofilm formation and gingival health in a group of young adults.

**Methods:**

The subjects were divided into two groups of 15 subjects each: control (A) and experimental group (B). The Turesky modification of Quigley-Hein plaque index was used to evaluate plaque scores while the presence or absence of gingival bleeding was used to determine gingival health. Measurements were done at baseline and at the end of the one-month trial period by one blinded examiner on six representative teeth. Group (A) maintained standardized oral health practices for the duration of the experiment. In addition, group (B) rinsed with a tablespoon of the provided sea salt mouth rinse for 30 seconds once in the morning and at night. After the 30-day trial period, subjects in both groups were reassessed as per baseline. Results. There were no statistically significant differences in the overall reduction from baseline in the mean plaque and gingivitis scores on all surfaces or on individual surfaces.

**Conclusion:**

Within the limitations of the study, rinsing with sea salt for thirty days did not affect the gingival and plaque scores in a group of young adults.

## 1. Introduction

Chronic gingivitis is a common inflammatory condition affecting a large population of the world and could lead to the development of periodontitis and eventual loss of teeth [1, 2]. As chronic gingivitis is initiated primarily as a result of biofilm accumulations, the standard practice of treatment is the removal of biofilm by mechanical disruption such as tooth brushing and flossing, proper plaque control instructions, and professional scaling and root planning [3]. However, maintaining a plaque-free environment may be difficult in patients with certain physical and mental disabilities or in the presence of local factors such as defective restorations, poor crown margins, or untreated cervical caries which could impede proper plaque control at these sites [4, 5]. Over the span of several years, there has been a plethora of plaque control aids, especially in the form of antiplaque or anti-inflammatory oral rinses and dentifrices that have been developed and used to facilitate the control of inflammation and supragingival plaque biofilm [6, 7]. A recent comprehensive study [8] systematically reviewed the existing literature on the efficiency of anti-inflammatory agents against chronic gingivitis, either as a solo or adjunctive therapy, and concluded that a beneficial effect did exist with the use of anti-inflammatory agents to manage the disease. However, the daily use of chemical agents should be advocated with caution due to potential side effects and taken into consideration the overall health of the patient and compliance to periodontal therapy [8]. As an example, the use of a well-known and effective antimicrobial, chlorhexidine gluconate (0.12%) could result in temporary loss of taste sensation, staining of teeth, restorations and mucosa, dryness and soreness of oral mucosa, and a slight increase in supragingival calculus [9]. There have also been reports of chlorhexidine-related allergies including anaphylaxis [10].

There is, therefore, a need for newer, cost effective, and tissue friendly rinses that can be used long term as an adjunct to standard oral hygiene methods. Salt water rinses have been traditionally recommended for use postoperatively, mainly after extractions, for periodontal and other oral infections, and for patients with alveolar osteitis [11–13].

The alleged beneficial effects of salt water rinses include promoting uncomplicated healing by inducing vasodilation and facilitating phagocytes to the site of injury, lowering the bacterial load by slightly alkalizing saliva, and acting as an astringent and as a bacteriostatic agent [9, 11]. Despite the wide use of salt water rinses, there is very little published information on the efficacy of this agent. The use of sea salt has been recently gaining popularity among the general population and is often promoted as being a healthier alternative to the less expensive table salt. Table salt is usually obtained from underground salt deposits and heavily processed to remove minerals, whereas sea salt is produced via evaporation of ocean or salt water lakes with very little processing [11, 14]. The purpose of this study was to evaluate a new sea salt mouth rinse containing xylitol and the antibacterial enzyme lysozyme on biofilm formation and gingival health in a group of young adults. Xylitol is a well-established nonsugar sweetener and an anticaries agent. The use of this polyol results in loosely adherent biofilms by reducing the amount of extracellular lipopolysaccharides and lipoteichoic acids, facilitating its easy removal by mechanical means [15]. Lysozymes are antibacterial proteins that hydrolyze the linkage between N-acetylmuramic acid and N-acetylglucosamine of peptidoglycan in the cell wall of Gram-positive  bacteria effectively limiting growth [16].

## 2. Materials and Methods

A sample of 30 participants, aged 20–26 years of age, were randomly selected (obtained from random.org) from among first and second year students currently enrolled at the College of Dentistry, University of Saskatchewan, Saskatoon, Canada. The inclusion criteria were as follows: being dentate, medically healthy, no orthodontic bands, and an anticipated ability to attend the two scheduled visits. Exclusion criteria included: a significant medical condition (including but not limited to, poorly controlled diabetes mellitus, rheumatic heart disease, or clinically significant heart murmur), pregnancy, and a recent history of, or ongoing, antibiotic therapy. All potential subjects were screened prior to the exam and selected as per the inclusion/exclusion criteria. The study was conducted in full accordance with ethical principles and with the approval of the University of Saskatchewan, Biomedical Research Ethics Board. Participants signed an informed consent form and were free to withdraw from the trial at any time. The experiment was designed as a randomized, single-blind study in which the subjects were randomly divided into two groups of 15 with group A being the control and group B the test.

The clinical examinations were conducted at the Dental clinic, College of Dentistry, University of Saskatchewan by one of the investigators (ET) who remained blind throughout the study period. This examiner was trained and calibrated to ensure the accuracy of the recordings. The calibration was done on five subjects not involved in the study. The Turesky-Gilmore-Glickman modification of Quigley-Hein plaque index [17] was used to evaluate plaque scores while the presence or absence of gingival bleeding on gentle probing the sulcus was used to determine gingival health. All measurements were done at baseline and at the end of the one-month trial period by the one blinded examiner on six representative teeth: 16, 21, 24, 36, 41, and 44. The plaque scores were assessed after disclosing with erythrosine red disclosing solution (1.4%) following gingival bleeding measurements. At the conclusion of the baseline measurements, the first group (A) was asked to maintain standardized oral health practices for the duration of the experiment in which they were required to brush their teeth for two minutes using the modified Bass technique, twice per day with the provided dentifrice and floss once daily using the spool method. Group B (test group) participants were also requested to brush and floss as above and then to rinse their mouth with a tablespoon of the provided sea salt mouth rinse (as per the manufacturer's guidelines) without any dilution, in the mouth for 30 seconds once in the morning and once before bed. Written instructions on the rinsing technique were also given, and each test participant was also advised to contact the authors if they noticed any untoward reactions following the rinse. No adverse events were reported. All participants were advised not to use any other oral hygiene products including antiseptic rinses during the period of study. After a 30-day trial period, subjects in both groups were reassessed as per baseline. The reduction from the baseline in the plaque and bleeding scores was calculated taking the difference between the scores at baseline and after the 30-day trial period for both control and test groups. The observed data, test scores (those who used sea salt and lysozyme) and control scores (those who did not use sea salt and lysozyme), were assumed to be sampled from populations with a normal distribution. Hence, the mean difference between the test and control groups' reduction in scores was analyzed using two independent samples *t*-test. The level of significance was set at *α* (alpha) = 0.05.

## 3. Results

The effects of rinsing the mouth with sea salt twice a day for a period of thirty days on plaque biofilm and bleeding on gentle probing the sulcus are shown in Table 1. There was no statistically significant difference in the overall reduction from baseline in relation to the mean plaque scores and the mean gingivitis scores for all surfaces when compared with the control group. The sample consisted of 17 females and 13 males.

Tables 2 and 3 show the effect of rinsing with sea salt on the individual surfaces (buccal, mesial, lingual, and distal) on plaque inhibition and gingivitis. There were no statistically significant differences on any of the four surfaces examined when compared with the control group.

Table 4 indicates the mean overall and site-specific baseline plaque and gingival scores. There was no statistically significant difference between the test and control cases, overall or on any of the four surfaces examined.

## 4. Discussion

Despite the lengthy practice of using saline solutions to manage selected oral conditions such as alveolar osteitis, acute mucosal lesions, and after minor oral surgical procedures, there are very little published clinical data attesting to its efficacy within the oral cavity. The present study involved the use of a mouth rinse consisting of sea salt, the antimicrobial enzyme lysozyme, and xylitol, a polyalcohol commonly used as a nonsugar sweetener, to determine its effect on plaque biofilm and gingivitis in a group of dental students.

The few clinical studies investigating the effect of rinsing with sea salt have been mostly carried out in the Philippines and India [12, 18]. Michel et al. [12] evaluated the effectiveness of sea salt rinse in street children of Manila, the Philippines, affected by mild-to-severe forms of periodontal disease. Most of these children were victims of abuse and neglect and were poor. The authors noted a decrease in gingival and periodontal indices at the end of the trial period during which each child rinsed with a solution containing 2.5 grams of sea salt in 20 ml of water. Mani et al. [18] investigated thirty adults with gingivitis attending a dental college in the state of Maharashtra, India and reported a significant reduction in all clinical parameters in subjects rinsing with sea salt for a period of three months compared to those who did not use the rinse.

The current study, however, did not demonstrate any statistically significant difference in plaque scores or gingivitis scores (bleeding on gentle probing the sulcus) between those who rinsed for thirty days with a solution containing sea salt, lysozyme enzyme, and xylitol with those who did not, in a group of dental students attending the College of Dentistry, Saskatoon, Canada. Dental students were utilized as subjects as they were present on site and were able to attend all scheduled examinations. It was also presumed that the students would be more compliant to following instructions specially pertaining to rinsing twice a day with the provided sea salt rinse than the population at large. However, dental students, in general, incline to have better oral hygiene and are supposedly better motivated than the general population, and consequently any changes in gingival bleeding and plaque inhibition before and after therapy may be assumed to have been slight and hence could have influenced the results. In order to minimize this limitation, the sample was randomly drawn from 1st and 2nd year students who tend to have a poorer oral hygiene than their senior counterparts [19].

Limitations of the present study also included the comparatively small sample size, the relatively short trial period, and possibly compliance issues within the test subjects who were provided bottles of the sea salt rinses to take home and were, therefore, unsupervised. Although written and verbal directions were provided, it is likely that some individuals may not have used the rinse as per instructions. In addition, the relatively short follow-up period of thirty days and the rinsing time of thirty seconds may have been not enough to produce a meaningful result and could be considered a limitation. This study was designed as a preliminary study to estimate the parameters for test and control measurements (plaque and gingivitis). Hence, it consisted of a convenience sample. Further, the time restraints (the study was conducted during the school term) and the cost involved to conduct the study using a larger population for an extend period of time also limited the sample size and the duration of the study. According to Connelly [20], extant literature suggests that a pilot study sample should be 10% of the sample projected for the larger parent study. Although this is not a straight forward issue [21], Isaac and Michael [22] and Hill [23] recommended 10–30 subjects for pilot studies. The full study, when conducted, will certainly involve a larger sample size and a study duration of 60–90 days.

## 5. Conclusion

Based on the current results, it appears that rinsing with a solution containing sea salt, xylitol, and lysozyme for thirty seconds, twice a day for a period of thirty days, has no significant benefit over brushing and flossing alone, on a sample of 30 dental students. However, considering some of the limitations of this pilot study and based on empirical information and traditional use of salt water rinses, further clinical studies must be attempted involving a larger sample size, subjects from the diverse population, and a longer trial period before any decisions could be made concerning its role as a therapeutic agent in the management of chronic gingivitis.

## Figures and Tables

**Table 1:** Overall reduction from baseline in the mean plaque scores and the mean gingivitis scores: all (buccal, mesial, lingual, and distal) surfaces.

Tooth surface	Test ± SD	Control ± SD	Difference ± SD	Paired *t* value	*P* value
Plaque	−0.12 ± 0.37	−0.11 ± 0.34	−0.01 ± 0.56	−0.096	0.925
Gingivitis	−0.01 ± 0.02	−0.01 ± 0.05	0.003 ± 0.06	0.186	0.855

**Table 2:** Reduction from baseline in the mean plaque scores: buccal, mesial, lingual, and distal surfaces.

Tooth surface	Test ± SD	Control ± SD	Difference ± SD	Paired *t* value	*P* value
Buccal	−0.21 ± 0.58	−0.25 ± 0.62	0.04 ± 0.84	0.205	0.840
Mesial	−0.19 ± 0.61	−0.15 ± 0.62	−0.03 ± 0.89	−0.144	0.887
Lingual	−0.02 ± 0.41	−0.09 ± 0.30	0.07 ± 0.61	0.421	0.680
Distal	−0.07 ± 0.57	0.07 ± 0.58	−0.13 ± 0.86	−0.601	0.558

**Table 3:** Reduction from baseline in the mean gingivitis scores: buccal, mesial, lingual, and distal surfaces.

Tooth surface	Test ± SD	Control ± SD	Difference ± SD	Paired *t* value	*P* value
Buccal	−0.02 ± 0.06	−0.01 ± 0.04	−0.01 ± 0.08	−0.564	0.582
Mesial	0.00 ± 0.00	−0.02 ± 0.12	0.02 ± 0.12	0.695	0.499
Lingual	−0.01 ± 0.08	−0.03 ± 0.09	0.02 ± 0.12	0.695	0.499
Distal	−0.01 ± 0.04	0.01 ± 0.02	−0.02 ± 0.11	−0.807	0.433

**Table 4:** Baseline mean overall and buccal, mesial, lingual, distal surfaces plaque scores, and gingivitis scores.

Tooth surface	Test ± SD	Control ± SD	*P* Value
*Plaque score*			
Overall	2.78 ± 0.31	2.79 ± 0.28	0.959
Buccal	1.57 ± 0.63	1.90 ± 0.62	0.156
Mesial	3.83 ± 0.47	3.63 ± 0.36	0.203
Lingual	2.25 ± 0.34	2.28 ± 0.44	0.879
Distal	3.48 ± 0.36	3.34 ± 0.33	0.298
*Gingivitis scores*			
Overall	0.01 ± 0.02	0.03 ± 0.06	0.225
Buccal	0.02 ± 0.06	0.01 ± 0.04	0.557
Mesial	0.01 ± 0.01	0.04 ± 0.09	0.104
Lingual	0.02 ± 0.06	0.04 ± 0.09	0.470
Distal	0.01 ± 0.04	0.03 ± 0.09	0.409

There are no significant differences (*P*>0.05) between the test and control cases baseline overall, and buccal, mesial, lingual, and distal surface plaque scores and gingivitis scores.
